# Fatty Acid Composition of Novel Host Jack Pine Do Not Prevent Host Acceptance and Colonization by the Invasive Mountain Pine Beetle and Its Symbiotic Fungus

**DOI:** 10.1371/journal.pone.0162046

**Published:** 2016-09-01

**Authors:** Guncha Ishangulyyeva, Ahmed Najar, Jonathan M. Curtis, Nadir Erbilgin

**Affiliations:** 1 Department of Renewable Resources, 4–42 Earth Sciences Building, University of Alberta, Edmonton, Alberta T6G2E3, Canada; 2 Department of Agriculture, Food and Nutritional Sciences, 3-60D South Academic Building, University of Alberta, Edmonton, Alberta T6G2P5, Canada; Natural Resources Canada, CANADA

## Abstract

Fatty acids are major components of plant lipids and can affect growth and development of insect herbivores. Despite a large literature examining the roles of fatty acids in conifers, relatively few studies have tested the effects of fatty acids on insect herbivores and their microbial symbionts. Particularly, whether fatty acids can affect the suitability of conifers for insect herbivores has never been studied before. Thus, we evaluated if composition of fatty acids impede or facilitate colonization of jack pine (*Pinus banksiana*) by the invasive mountain pine beetle (*Dendroctonus ponderosae*) and its symbiotic fungus (*Grosmannia clavigera*). This is the first study to examine the effects of tree fatty acids on any bark beetle species and its symbiotic fungus. In a novel bioassay, we found that plant tissues (hosts and non-host) amended with synthetic fatty acids at concentrations representative of jack pine were compatible with beetle larvae. Likewise, *G*. *clavigera* grew in media amended with lipid fractions or synthetic fatty acids at concentrations present in jack pine. In contrast, fatty acids and lipid composition of a non-host were not suitable for the beetle larvae or the fungus. Apparently, concentrations of individual, rather than total, fatty acids determined the suitability of jack pine. Furthermore, sampling of host and non-host tree species across Canada demonstrated that the composition of jack pine fatty acids was similar to the different populations of beetle’s historical hosts. These results demonstrate that fatty acids composition compatible with insect herbivores and their microbial symbionts can be important factor defining host suitability to invasive insects.

## Introduction

Herbivorous insects depend on the nutritional composition of their host plants and have developed several mechanisms to consume host plant resources and to cope with their defenses [1–3]. Thus, their ability to attack and reproduce may depend on host plant quality, characterized by primary (availability of nitrogen and carbon-based compounds, such as carbohydrates and lipids) and secondary (defensive compounds) compounds of plants [4–8]. In general, studies focusing on plant-insect interactions have mainly focused on the secondary compounds of plants [4, 9–11], while the role of plant nutrients remains poorly understood despite their abundance and importance to insect herbivores and their symbionts [5, 8, 12, 13].

Plants are rich in fatty acids, which are the major components of lipids. The most common fatty acids have hydrocarbon chain lengths ranging from 16 to 22 carbon atoms. Depending on the chain lengths and degree of saturation, fatty acids are broadly classified either as saturated or unsaturated [14]. Three aspects of plant fatty acids are particularly relevant to investigate their effects on insect herbivores. First, some plant fatty acids are required by insects to sustain growth, development, and mate recognition [15–17]. For example, many lepidopterans (butterflies and months) acquire linoleic (18:2ω6) or alpha-linolenic (18:3ω3) acids from host plants to sustain their growth [15]. Likewise, mountain pine beetle (MPB, *Dendroctonus ponderosae* Hopkins) (Coleoptera: Curculionidae) produces *exo*-brevicomin pheromone from a fatty acyl-derived precursor [18] which either originates from trees or produced *de novo* [19]. Second, plants synthesize many fatty acid derivatives, notably jasmonic acids which are biosynthesized from an alpha-linolenic acid precursor and regulate plant defense responses to insect herbivores [20]. Third, despite a large literature examining the roles of fatty acids in conifers [21–24], relatively few studies have tested the effects of fatty acids on forest insects and their microbial symbionts [25–28]. Particularly, if fatty acids facilitate colonization of novel plants by invasive forest insects have never been studied.

Here we studied the roles of fatty acids in the suitability, and thus colonization, of the novel host jack pine for the invasive mountain pine beetle (MPB, *Dendroctonus ponderosae* Hopkins) (Coleoptera: Curculionidae). The mountain pine beetle is the most damaging native insect mortality agent of pine forests of western North America [29]. When new brood beetles emerge from the trees in which they developed and overwintered, they disperse and seek new suitable trees during early summer. When a tree is selected, beetles may overcome tree defenses by engaging pheromone-mediated mass attacks and inoculating trees with their symbiotic fungi including *Grosmannia clavigera* [30]. Symbiotic fungi can facilitate beetle nutrition either directly by serving as a food source (nitrogen and sterols), or indirectly by concentrating nutrients in host phloem tissue [30, 31]. Larvae construct galleries as they feed on phloem and symbiotic fungal hyphae, and overwinter as late instars. Pupation occurs in the spring, followed by development into adults in early summer. There is commonly one generation per year.

Mountain pine beetle colonizes all pine (*Pinus*) species within its historical range including lodgepole (*P*. *contorta*), ponderosa (*P*. *ponderosa*), and limber (*P*. *flexilis*) pines [32]. Host and geographical range of MPB had historically been limited by climatic barriers; however recently MPB has overcome these barriers and become invasive in the jack pine (*P*. *banksiana*) forests in Alberta [33, 11]. Jack pine is considered a novel host of MPB and is a major component of the boreal forest, which extends from Alberta to eastern Canada, overlapping with red pine (*P*. *resinosa*) in the Great Lakes and northeastern regions of the USA. Although the roles of plant secondary metabolites, mainly monoterpenes, in jack pine suitability to MPB have been described [11], we have no knowledge of whether jack pine fatty acids also affect host suitability.

Our goal was to determine whether composition of fatty acids has facilitated colonization of jack pine by MPB and its symbiotic fungus. This is the first study to examine the effects of tree fatty acids on the larval survival of any bark beetle species and growth of its symbiotic fungus. First, we characterized the fatty acid composition in beetle’s historical (lodgepole, limber, and ponderosa pines), novel (jack pine), occasional (white spruce, *Picea glauca*), and potential (red pine and scots pine, *P*. *sylvestris*) hosts. White spruce was selected as an occasional host because MPB occasionally attack and produce broods on this species during outbreaks [34]. Red and scots pines were included as potential hosts because they are commonly found in jack pine forests although the latter is native to Europe. Second, we selected lodgepole and jack pine trees that co-occur in Alberta and conducted bioassays to determine the effects of their fatty acids on MPB and *G*. *clavigera*. To illustrate the consequences of incompatibility of fatty acids with MPB and the fungus, we intended to select one pine species as a non-host, but such species does not exist as MPB can attack all pines (native and exotic) in its natural range [32]. Thus, aspen (*Populus tremuloides*) was selected as a non-host. Although aspen is phylogenetically distanced from known MPB hosts, our aim was to validate the consequences of incompatible fatty acids on beetle survival and fungal growth. Otherwise, we did not intend to compare the suitability of aspen with that of known MPB hosts, nor disregard that factors other than fatty acids can distinguish aspen from pines. Effects of fatty acids on survival of MPB larvae were examined using a novel bioassay consisting of tree tissues amended with synthetic fatty acids at concentrations simulating fatty acid composition in lodgepole pine, jack pine, or aspen. Similarly, the growth of *G*. *clavigera* was examined in media amended with lipid fractions or synthetic fatty acids at concentrations representative of these tree species.

Our research objectives were to: (1) characterize the fatty acid composition in historical, occasional, potential, and novel hosts, as well as aspen, (2) quantify the major fatty acids present in lodgepole and jack pines and aspen, and (3) determine the toxic effects of fatty acids of lodgepole pine, jack pine, or aspen on MPB larval survival and *G*. *clavigera* growth.

## Materials and Methods

### Does fatty acid composition vary among tree species?

#### Sample collection

**Fig 1:**
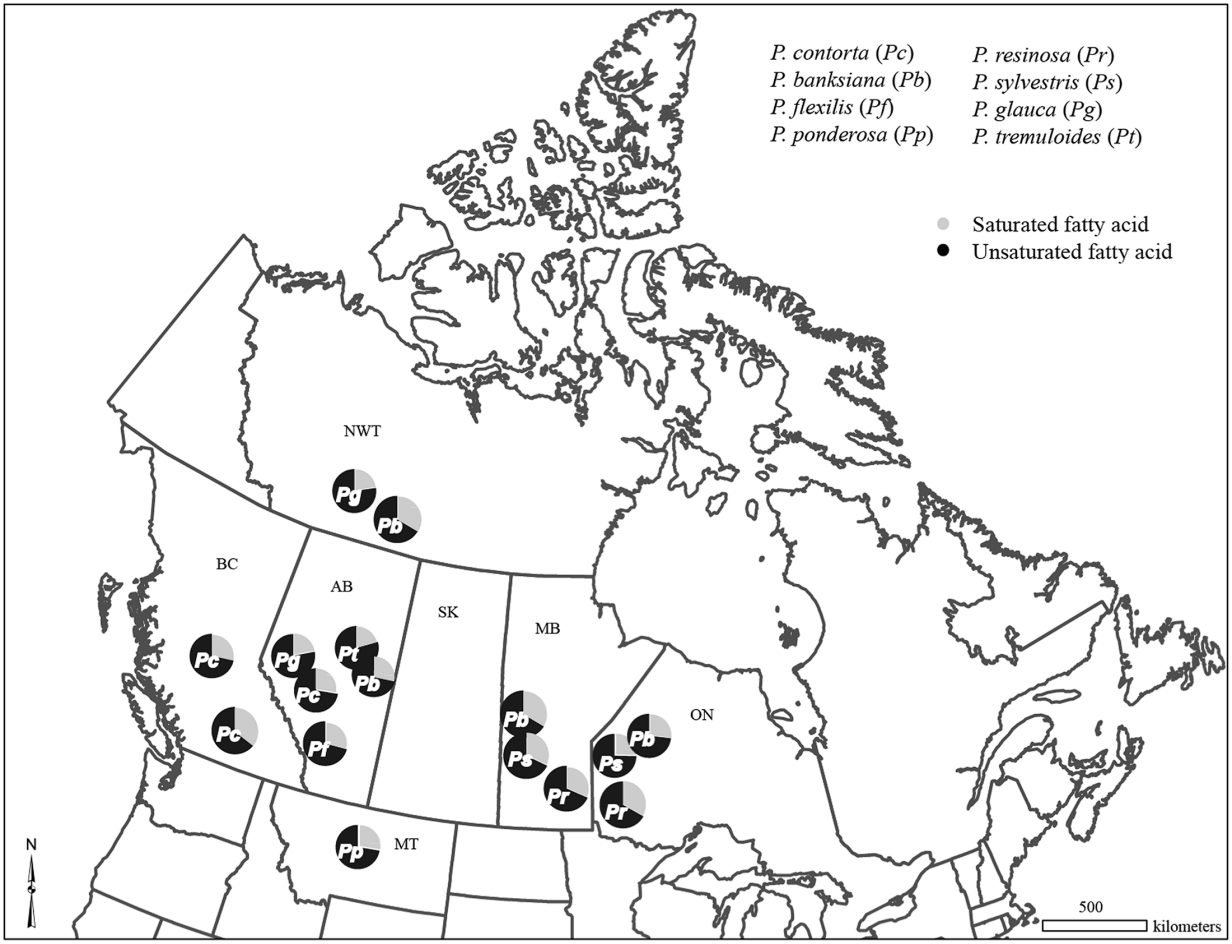
Mean ratios of saturated and unsaturated fatty acids (μg/g of dry weight of phloem) of various tree species sampled across North America. Abbreviated letters denote sample locations: AB = Alberta, BC = British Columbia, MB = Manitoba, ON = Ontario, NWT = Northwest Territories, MT = Montana. In each pie chart, the ratios are shown along with two-letter abbreviations denoting tree species: Pc: *Pinus contorta*, Pb: *P*. *banksiana*, Pr: *P*. *resinosa*, Ps: *P*. *sylvestris*, Pb: *P*. *ponderosa*, Pf: *P*. *flexilis*, Pg: *Picea glauca*, and Pt: *Populus tremuloides*.

We collected phloem samples from eight tree species (*P*. *contorta*, *P*. *banksiana*, *P*. *flexilis*, *P*. *ponderosae*, *P*. *resinosa*, *P*. *sylvestris*, *P*. *glauca*, and *P*. *tremuloides*) in Canada and the US (Fig 1). Diameters of trees were 25–30 cm at breast height (1.40 cm) where samples were collected and trees were 60–70 years old. Phloem samples, each 5 cm x 5 cm in size, were removed from bark on both south and north aspects of each tree using a hammer and chisel. Samples were kept on dry ice and shipped to the University of Alberta. For some species (*P*. *contorta*, *P*. *banksiana*, *P*. *resinosa*, *P*. *sylvestris*, *P*. *glauca*), we collected phloem samples from multiple locations. To compare the effects of fatty acids on beetle larvae and the fungus, additional phloem samples were collected from lodgepole pine, jack pines, and aspen in Alberta in summer 2013. Overall, 12 to 45 trees per species were sampled at each location with a total of 430 samples. We were not required to obtain any specific permit for any of sampling activities and the field studies did not involve any endangered or protected species.

#### Fatty acid extraction and chemical analysis

Upon receiving, samples were freeze dried for 72 hr (Labconco Corp., USA), ground (Tissue Lyser II, QIAGEN Corp., Germany), and stored at -40°C until extraction. We put 100 mg of ground sample into a 50 mL glass centrifuge tube for extraction following the method described by Curtis et al. [35]. Briefly, samples were mixed with 1 mL of recovery standard (M_C19_), 1 mg/g of trinonadecanoin solution in toluene (Nu-Chek Prep Inc., MN, USA) in the glass tube. We added 2 mL of toluene and 6 mL of a freshly prepared acetyl chloride solution (10% acetyl chloride in methanol) to the tube, which was then vortex mixed for 30 sec and placed into water bath at 80°C for 2 h. The tube was left to cool at room temperature for 15 min before it was vortex mixed for 30 sec, after which time 10 mL of 6% sodium carbonate in deionized distilled water was added to the tube. To separate the toluene containing fatty acid methyl esters from the mixture, we centrifuged the mixture at 1,100 rpm for 15 min, collected the separated organic layer, and transferred to a 15 mL glass vial containing 1 g of sodium sulfate, which absorbed residual water in the solution. The mixed solution was vortexed for 30 sec. Then, we added 1 mL of the toluene extract to 0.2 mL of a standard solution (M_C23_), containing 0.7 mg methyl tricosanoate (Nu-Chek Prep Inc.). The resulting solution was placed in a 2 mL glass vial for chemical analysis.

We injected 1 μL of sample in splitless mode into a Gas Chromatograph-Mass Spectrometer (Agilent Tech., CA, USA) equipped with a capillary column HP-88 (60 m length, 0.25 μm film, 250 μm I.D.) and with carrier gas of helium at 1.0 mL min^-1^ flow rate. The temperature program started at 75°C and increased at 40°C min^-1^ to 145°C, held for 2 min, then increased at 10°C min^-1^ to 205°C, held for 5 min, and then increased at 20°C min^-1^ to 250°C and held for 1 min. The recovery coefficient was around 73% as per use of M_C19_ standard. We identified 21 fatty acid methyl esters from seven species (*P*. *ponderosa* was not included because we have a relatively small tissue to analyze) (Table 1). We discarded peaks with a mean relative proportion of less than 0.1% from further analyses.

**Table 1:** Fatty acid methyl ester (ME) profiles identified from the phloem of *Pinus contorta* (Pc), *P*. *banksiana* (Pb), *P*. *resinosa* (Pr), *P*. *sylvestris* (Ps), *P*. *flexilis* (Pf), *Picea glauca* (Pg), and *Populus tremuloides* (Pt). C:D (n-x) indicate the number of carbon atoms, double bonds in the straight chain of fatty acids, and name of individual compound, respectively. (+) and (−) denote presence and absence of a particular fatty acid in each species.

Compound name	Common name	Abbreviations used in text	C:D (n-x)	Pc	Pb	Pr	Ps	Pf	Pg	Pt
Pentanoic acid, *4-oxo*, ME	Levulinic acid	LvA	4 = oxo-5:0	+	+	+	+	+	+	+
Cyclopentanetridecanoic acid ME	Cyclopentanetridecanoic acid		-	+	+	+	+	+	−	+
Hepta-*2*,*4*-dienoic acid ME	Hepta-2,4-dienoic acid		7:2	−	−	−	−	−	−	+
Nonanoic acid ME	Pelargonic acid		9:0	−	−	+	−	−	−	+
Decanoic acid ME	Capric acid		10:0	+	+	−	−	−	−	−
Pentadecanoic acid ME	Pentadecanoic acid	PDA	15:0	+	+	+	+	+	+	+
Hexadecanoic acid ME	Palmitic acid	PA	16:0	+	+	+	+	+	+	+
Hexadecanoic acid,14-methyl-, ME	14-Hexadecanoic acid		14methyl-16:0	+	−	−	−	+	+	−
Heptadecanoic acid, *16*-methyl-, ME	16-Heptadecanoic acid		16methyl-17:0	−	+	−	−	−	−	−
Octadecanoic acid ME	Stearic acid	SA	18:0	+	+	−	−	+	+	+
*9*-Octadecenoic (*Z*) ME	Oleic acid	OA	18:1 (n-9)	+	+	+	+	+	+	+
*9*,*12*-Octadecadienoic acid (*ZZ*) ME	Linoleic acid	LA	18:2 (n-6)	+	+	+	+	+	+	+
*8*,*11*-Octadecadienoic acid ME	8,11-Octadecadienoic acid		18:2 (n-7)	+	−	+	−	+	+	−
*9*,*12*,*15*-Octadecatrienoic (*ZZZ*) ME	Alpha-linolenic acid	ALA	18:3 (n-3)	+	+	+	+	+	+	+
*6*,*9*,*12*-Octadecatrienoic acid (ZZZ) ME	Gamma-linolenic acid	GLA	18:3 (n-6)	+	+	+	+	+	+	+
Eicosanoic acid ME	Arachidic acid		20:0	+	+	−	+	+	−	−
*11*,*14*-Eicosadienoic acid (*ZZ*) ME	Eicosadienoic acid	EDA	20:2 (n-6)	+	+	+	+	+	+	+
*11*,*14*,*17*-Eicosatrienoic acid ME	Eicosatrienoic acid		20:3(n-3)	+	+	−	+	+	−	−
*5*,*8*,*11*,*14*-Eicosatetraenoic (all *Z*) ME	Arachidonic acid	ARA	20:4 (n-6)	+	+	+	+	+	+	+
Docosanoic acid ME	Behenic acid	BA	22:0	+	+	+	+	+	+	+
*13*,*16*-docosadienoic acid ME	Docosadienoic acid		22:2 (n-6)	+	+	+	+	+	+	−

Eleven fatty acid methyl esters were common among all tree species and made up 95% of total fatty acids based on peak areas of individual fatty acid methyl esters relative to the total peak areas of total ion chromatograms. We quantified these 11 fatty acids using the following standards: methyl pentadecanoate, methyl palmitate, methyl stearate, methyl oleate, methyl linolenate, methyl gamma linolenate, methyl 11,14-eicosadienoate, methyl arachidonate, methyl behenate, and methyl linoleate (Nu-Chek Prep Inc.), and methyl levulinate (Sigma-Aldrich). These compounds had a purity of >99%. We categorized levulinic acid as a saturated fatty acid because it is used in fatty acid synthesis.

#### Data analyses

We used R 3.0.2, R Development Core Team [36] for all analyses. Samples representing a tree species in each location were pooled. After square-root transformations to meet the assumptions of normality and homogeneity of variance, we conducted ANOVA to test differences in the total concentrations of fatty acids among eight tree species and the individual fatty acid concentrations among lodgepole pine, jack pine, and aspen. We performed Tukey’s Honestly Significant Difference (HSD) to adjust for multiple comparisons. Moreover, we used a canonical discriminant analysis (“candies” package in R) with group centroids, 95% confidence interval, and vectors representing fatty acid concentrations to test whether compositions of fatty acids among eight tree species vary. We conducted similar analysis among lodgepole pine and jack pine, and aspen in axes Can1 and Can2 and reported the proportions of total saturated and unsaturated fatty acids of each species at each sampling collection point.

### Do fatty acids of tree species differentially influence growth of *G*. *clavigera*?

To determine if *G*. *clavigera* can grow in media amended with lipid fractions or synthetic fatty acids at concentrations representative of lodgepole pine, jack pine, or aspen, we inoculated the fungus into the media. The fungus that was originally collected from Fox Creek, Alberta was isolated from adult and infected wood adjacent to beetle galleries in mature pine trees as described in Goodsman et al. [31]. In our preliminary trials, we first incorporated ground lodgepole pine substrate in Petri Dishes as a medium to observe the fungal growth. But we did not observe any fungal growth on any of the plates because probably nutritional component of pine substrate was not sufficient for the fungus. For this reason, an artificial medium (Bacto^™^ Agar, Becton Dickinson and Company Sparks, MD, USA) was used in our experiments.

#### Lipid extraction and fatty acid purification

In July 2013, we cut 4 trees (25–30 cm dia. at breast height) per species (lodgepole pine: Hinton, Alberta, 53°45.925'N, 118°22.298'W; jack pine and aspen: Lac La Biche, Alberta, 54°16.964'N, 111°37.251'W). The bolts were kept at 4°C until use. Phloem and sapwood with a depth of 1 cm from bolts were polled and placed in an oven at 60°C for 3 d. The dried phloem and sapwood samples were separately ground in Wiley Mill with 1 mm mesh screen and then were mixed (9:1 ratio of phloem to sapwood) together and extracted for lipids, following methods by Fischer and Höll [37]. Briefly, in a 50 mL centrifuge tube, 1.5 g of a ground tissue was mixed with 40 mL of a solvent (chloroform/methanol, 2:1 v/v, at 99.9% purity, Sigma-Aldrich). The resulting solution was mixed in a vortex for 30 sec and then left to rest at room temperature for 3 min. The tube was centrifuged for 5 min at 2,500 rpm and the resulting separate organic layer was collected into a new centrifuge tube. This process repeated twice. The third extraction was similar to the first two, but with a different solvent ratio (chloroform/methanol, 1:2 v/v). In all cases, after extraction and centrifugation, the resulting separated organic layers were combined. We added 4.5 mL of 3.6 μm CaCl_2_ solution to the tube and then shook it for 5 sec. The new solution was then centrifuged for 3 min at 3,000 rpm and the resulting layer containing chloroform (15 mL) with lipids was taken for further purification in a solid phase extraction cartridge (HF Mega BE-NH2, sorbent weight = 10g, tube volume = 60mL, Agilent Tech.).

The solid phase extraction cartridge was placed on the top of a vacuum Elute 20manifold (Agilent Tech.). We used 120 mL of hexane to condition the cartridge and then processed 150 mL of lipid extract from the phloem-sapwood mixture. We used 150 mL of chloroform/propanol (2:1 v/v) in order to retain only free fatty acids and phospholipids in the matrix. In order to elute free fatty acids, a 100 mL solution of 2% acetic acid in diethyl ether (both at 99.9% purity, Fischer Sci.) was used. The phospholipids in the cartridge were retained and the eluent from the cartridge was collected into a glass vial and dried under a stream of N_2_ gas. The evaporate was then weighted. The purified total ‘natural’ extracts were as kept at -80°C until use.

After lipid extraction and purification of tissues, the remaining fat-free solid tissue samples were dried in the oven at 70°C for 3 d and presented as media to MPB larvae within the tubes (see *Media preparation and rearing tube experiment* section below).

#### Media preparation

Lipid fractions and synthetic fatty acids were amended with the media in two separate experiments. In one experiment, total purified lipid fractions (mainly free fatty acids but also contains some triglycerides) were prepared based on their natural concentrations (wet phloem) in each tree species: lodgepole pine (249.22 μg/mg), jack pine (48.31 μg/mg), and aspen (125.84 μg/mg). Total purified lipid fractions, 20 mL of sterilized distilled water, and 10 μL of Tween^®^ 20 were mixed together and sonicated for 20 min. For the media preparation, 3 g of Bacto^™^ Agar was mixed with 180 mL of distilled water in a 1L of glass flask. The media was autoclaved at 121°C for 20 min which was then cooled at room temperature for 35–40 min. Homogenized lipid fractions was added into the media and then the whole mixture was shaken for 1 min and poured into sterilized Petri dishes (100x15mm) (Fischer Sci.). Plates were ready for fungal inoculation 24 h later. A plug of culture containing live *G*. *clavigera* propagules was inoculated into plates using a cork borer (5 mm in dia) (n = 15) and the plates were sealed with Parafilm and kept at room temperature for 4 wks.

**Fig 2:**
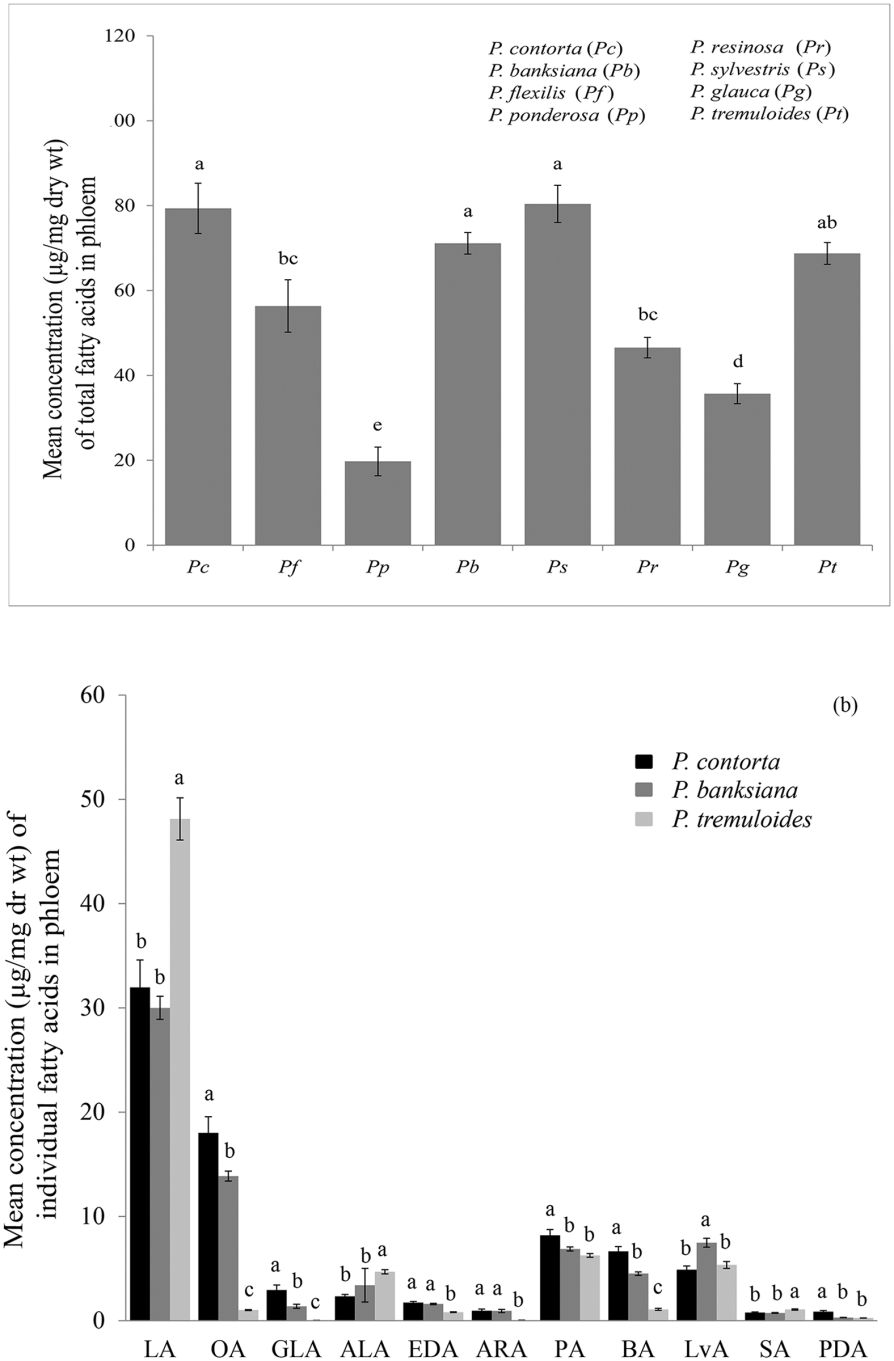
Differences in the mean (± SE) concentrations of fatty acids among tree species. An ANOVA and Tukey’s HSD were performed for multiple comparisons among species. Different letters after means indicate significant difference among species at α = 0.05. **(A)** Mean concentrations of total fatty acids among Pc (*Pinus contorta*, n = 25), Pf (*P*. *flexilis*, n = 22), Pp (*P*. *ponderosa*, n = 31), Pb (*P*. *banksiana*, n = 25), Ps (*P*. *sylvestris*, n = 25), Pr (*P*. *resinosa*, n = 25), Pg (*Picea glauca*, n = 25), and Pt (*Populus tremuloides*, n = 25). **(B)** Mean concentrations of individual fatty acids among *P*. *contorta*, *P*. *banksiana*¸ and *P*. *tremuloides*. Acronyms for individual fatty acids were shown in Table 1. F_2,72_ and P-values are: PA: 5.29, <0.001; BA: 141.72, <0.001; LvA: 12.7, <0.001; PDA: 32.65, <0.001; SA: 9.01, <0.001; LA: 21.95, <0.001; OA: 215.67, <0.001; GLA: 62.86, <0.001; ALA: 8.21, <0.001; EDA: 53.68, <0.001; ARA: 23.22, <0.001.

Since the results of experiments with the lipid fractions were inconclusive (see results in *Fungal growth* section below), the same experiment was repeated with synthetic fatty acids at concentrations representative of each tree species (Fig 2B, S1 Table). Individual fatty acids were either combined to make a specific blend of total saturated or unsaturated fatty acids that simulated composition of each species or used individually. The amounts amended were calculated based on the concentrations of individual fatty acids from each species on a dry weight basis. The fungal growth (mm^2^ area covered) on each plate was compared among tree species after 4 wks.

#### Data analyses

To determine the relationship between lipid fractions or synthetic fatty acids (total saturated or unsaturated or individual) and *G*. *clavigera* growth, we used ANOVA after square-root transformations to meet the assumptions of normality and homogeneity of variance. Tukey’s HSD was performed for multiple comparisons [36].

### Do fatty acids of tree species differentially influence MPB larval survival?

To test the biological significance of fatty acids on MPB larval survival, a novel bioassay was conducted using defined concentrations of synthetic fatty acids representative of lodgepole pine, jack pine, or aspen. We either combined individual fatty acids to make a specific blend of total saturated or unsaturated fatty acids that simulated the composition of each tree species or used them individually. As indicated above, supernatant remaining after lipid extraction and purification was presented as a medium to beetle larvae inside the tubes.

#### Media preparation and rearing tube experiment

A new rearing method was used to determine if fatty acids affect larval survival as this technique allowed us to manipulate the content of the beetle-rearing environment. The technique was developed by Myrholm and Langor [38]. Briefly, we plugged one end of a glass tube (10 cm long, 5 mm OD, 3.6 mm ID, Kimax Standard, Fischer Sci.) with an absorbent cotton wool, filled the tube with lightly compressed 0.45 g (dry) of ground tree substrate (9:1 ratio of phloem and sapwood) using a sterile funnel, and plugged in the opposite end of the tube with cotton wool. A thin strip of Parafilm was wrapped around tube ends and a pipette filter tip (200 μL) lightly filled with glass wool was tightly fitted to minimize contamination while allowing air circulation inside the tube. The entire unit was then autoclaved twice at 121°C for 30 min and kept at room temperature.

A single MPB egg was placed in each tube. To place individual eggs, the filter tip and cotton plug from one end was removed, and a small depression (5 mm deep) was made in the substrate close to one end of the tube using a sterile 200 μL pipette tip. Under a dissecting microscope, eggs were gently placed into the depression using a 200 μL pipette tip. Fungal cultures that were used to test the effects of fatty acids on the growth of *G*. *clavigera* above were placed into the same depression without putting pressure on the egg. Finally, fatty acids mixed with 10 μL of Tween^®^ 20 (Sigma-Aldrich) were injected to the tubes (n = 15). Cotton plugs and pipette tips were replaced. All tubes were placed into sterile covered trays and incubated at 24°C in darkness for 70 d. Larval survival was quantified at the end of experiment. All this work was conducted under a laminar flow hood using sterile technique.

Beetle eggs were obtained from recently infested lodgepole pine bolts in the laboratory. On each bolt, eggs were carefully removed from beetle galleries and placed them onto sterile, moistened filter paper in Petri dishes. Eggs were surface sterilized in a modified White’s Solution [30], and subsequently stored them at 4°C for up to 7 d on sterile, moistened filter. Eggs that had no discoloration or evidence of deflation were selected prior to embryo development [39].

In the first experiment, we either amended lodgepole pine substrate with the total saturated or unsaturated synthetic fatty acids at defined concentrations representative of each of the above three species, or individually amended the substrates from these species with the total saturated or unsaturated synthetic fatty acids at concentrations representative of only lodgepole pine. In the second experiment, based on the results of fungal growth (described above), the bioassays were conducted using defined concentrations of five fatty acids (behenic, palmitic, oleic, alpha-linolenic, linoleic) that simulated the composition of each tree species and amended with the species-specific substrates (e.g. jack pine substrate was amended with alpha-linolenic acid of jack pine). The amounts injected into each tube were calculated based on the concentrations of individual fatty acids quantified for each tree species on a dry weight basis (Fig 2, S2 Table).

#### Data analyses

The proportions of the larval survival was calculated in either lodgepole pine substrate, amended with total saturated or unsaturated fatty acids at defined concentrations of lodgepole pine, jack pine or aspen, or the substrate of these tree species amended with total saturated or unsaturated fatty acids representative of lodgepole pine. The proportions of larval survival were also assessed for individual fatty acids representing each species. To test the effects of fatty acids on larval survival Permutational ANOVA and Tukey’s HSD for multiple comparisons (lmPerm package in R version 3.0.2 “Frisbee Sailing” [36] was performed.

## Results

Using the same method to analyze individual chemicals in the trees, we confirmed that our methanol:chloroform extraction yielded mainly free fatty acids and potentially some triglycerides. A total of 21 fatty acids were identified from seven tree species (*P*. *ponderosa* was not included). The list of compounds along with their chemical names was reported in Table 1. Eleven of these (4-oxo-levulinic, pentadecanoic (15:0), palmitic (16:0), gamma-linolenic (18:3ω6), oleic (18:1ω9), linoleic (18:2ω6), alpha-linolenic (18:3ω3), stearic (18:0), behenic (22:0), arachidonic (20:0), and eicosadienoic (20:2ω6)) were found in all species, while others were unique to one or two species. For simplicity, hereafter we will refer to these 11 fatty acids with their common names.

Ratios of unsaturated/saturated fatty acids were consistent among tree species, and all species in all locations contained more unsaturated than saturated fatty acids, ranging from 0.65 to 0.79 of total fatty acids (Fig 1). Notably, lodgepole and jack pines contained more fatty acids in northern latitudes than those in more southern latitudes (Table 2).

**Table 2:** Mean (± SE) concentration of major fatty acids (μg/g of dry weight of phloem) from *Pinus contorta*, *P*. *banksiana*, *P*. *resinosa*, *P*. *sylvestris*, *P*. *ponderosa*, *P*. *flexilis*, *Picea glauca*, and *Populus tremuloides* sampled across North America. Acronyms for individual fatty acids were shown in Table 1. (-) denotes absence.

Tree species	Location (n)	LA	OA	PA	BA	LvA	GLA	ALA	EDA	ARA	PDA	SA	Total
***P*. *contorta***	Alberta (**25**)	31.98 (2.61)	18.02 (1.53)	8.18 (0.56)	6.65 (0.46)	4.89 (0.35)	2.95 (0.47)	2.34 (0.17)	1.74 (0.11)	0.95 (0.17)	0.86 (0.11)	0.78 (0.06)	79.34 (5.91)
	Central BC (**30**)	24.99 (1.75)	13.37 (1.00)	5.62 (0.43)	4.65 (0.40)	7.37 (0.72)	5.02 (0.58)	2.55 (0.20)	1.73 (0.14)	1.06 (0.15)	1.22 (0.13)	0.67 (0.08)	68.26 (4.63)
	Southern BC (**35**)	11.41 (0.67)	5.67 (0.37)	3.01 (0.15)	2.48 (0.14)	5.82 (0.40)	1.96 (0.25)	1.19 (0.07)	1.07 (0.05)	0.54 (0.08)	0.38 (0.04)	0.10 (0.03)	33.63 (1.55)
***P*. *banksiana***	Alberta (**25**)	30.00 (1.11)	13.87 (0.48)	6.88 (0.20)	4.52 (0.17)	7.49 (0.43)	1.38 (0.20)	3.40 (1.61)	1.61 (0.07)	0.93 (0.14)	0.29 (0.01)	0.74 (0.04)	71.12 (2.54)
	Northwest Territories (**12**)	47.76 (4.25)	21.37 (1.76)	10.14 (0.69)	8.30 (0.69)	18.01 (1.66)	1.01 (0.20)	2.38 (0.22)	2.79 (0.27)	1.49 (0.34)	0.45 (0.04)	1.33 (0.14)	115.03 (8.99)
	Manitoba (**40**)	27.03 (1.01)	13.15 (0.67)	6.19 (0.24)	5.42 (0.19)	10.62 (0.92)	1.03 (0.11)	1.84 (0.07)	1.63 (0.08)	0.78 (0.10)	0.26 (0.01)	0.78 (0.04)	68.74 (2.63)
	Ontario (**45**)	43.00 (2.37)	21.10 (1.27)	8.69 (0.49)	8.33 (0.45)	7.72 (0.48)	1.22 (0.11)	2.58 (0.15)	2.75 (0.16)	1.52 (0.19)	0.31 (0.02)	1.15 (0.07)	98.36 (5.34)
***P*. *resinosa***	Manitoba (**24**)	17.58 (0.96)	10.64 (0.69)	1.81 (0.09)	4.09 (0.23)	8.43 (0.48)	0.19 (0.04)	1.77 (0.10)	1.26 (0.07)	0.67 (0.09)	0.07 (0.02)	—	46.53 (2.40)
	Ontario (**25**)	16.31 (0.72)	9.03 (0.50)	1.92 (0.08)	3.88 (0.15)	8.36 (0.49)	0.16 (0.01)	1.76 (0.08)	1.22 (0.07)	0.37 (0.07)	0.05 (0.01)	0.05 (0.03)	43.10 (1.81)
***P*. *sylvestris***	Manitoba (**25**)	32.86 (2.04)	16.23 (1.25)	3.79 (0.21)	5.94 (0.37)	15.69 (1.26)	0.96 (0.13)	2.08 (0.13)	1.68 (0.10)	0.98 (0.20)	0.19 (0.02)	—	80.40 (4.38)
	Ontario (**25**)	46.73 (2.22)	23.37 (1.47)	4.58 (0.18)	7.07 (0.26)	13.35 (0.65)	0.80 (0.09)	2.98 (0.15)	2.16 (0.08)	0.96 (0.19)	0.16 (0.03)	—	102.15 (3.91)
***P*. *flexilis***	Alberta (**22**)	23.79 (2.46)	10.17 (91.13)	6.86 (0.70)	5.28 (0.54)	2.56 (0.39)	2.79 (0.82)	1.66 (0.20)	2.05 (0.20)	0.33 (0.09)	0.86 (0.16)	0.88 (0.10)	57.23 (5.85)
***P*. *ponderosa***	Montana (**31**)	3.29 (1.00)	1.90 (0.53)	1.50 (0.21)	1.86 (0.35)	0.38 (0.14)	3.96 (1.70)	0.52 (0.08)	2.27 (0.38)	1.45 (0.45)	0.56 (0.16)	0.13 (0.04)	16.24 (2.45)
*P*. *glauca*	Alberta (**25**)	16.12 (1.04)	5.16 (0.60)	3.38 (0.23)	3.12 (0.26)	1.02 (0.18)	2.63 (0.20)	1.74 (0.09)	1.77 (0.10)	0.41 (0.12)	0.33 (0.05)	0.03 (0.02)	35.70 (2.36)
	Northwest Territories (**25**)	14.38 (0.85)	3.70 (0.26)	2.30 (0.18)	3.44 (0.23)	1.40 (0.10)	3.20 (0.48)	1.33 (0.06)	2.46 (0.19)	1.00 (0.29)	0.42 (0.07)	—	33.64 (1.43)
***P*. *tremuloides***	Alberta (**25**)	48.12 (2.03)	1.02 (0.05)	6.26 (0.18)	1.09 (0.10)	5.35 (0.33)	0.03 (0.004)	4.69 (0.20)	0.81 (0.03)	0.03 (0.02)	0.26 (0.01)	1.07 (0.06)	68.73 (2.59)

### Comparisons of fatty acid profiles among tree species

The concentrations of fatty acids were significantly different between the eight tree species (ANOVA, Fig 2A). With the exception of aspen and jack pine, the mean total concentration of fatty acids in lodgepole pine and scots pines were higher than those in the remaining four species. There was no difference among lodgepole, scots and jack pines, and aspen. Likewise, the mean total concentrations of fatty acids in red pine and white spruce were similar. Ponderosa pine had the lowest mean total concentration of fatty acids among tree species.

Comparisons of mean concentrations of 11 fatty acids indicated differences among lodgepole and jack pines and aspen (ANOVA, Fig 2B, Table 2). Five fatty acids including the most abundant linoleic acid did not vary between the two pine species. In contrast, 10 of 11 fatty acids differed between lodgepole pine and aspen, and 9 of 11 were different between jack pine and aspen. Aspen contained the highest concentrations of linoleic, alpha-linolenic, and stearic acids and the lowest concentrations of oleic, gamma-linolenic, eicosadienoic, arachidonic, and behenic acids. Notably, linoleic acid was the most abundant fatty acid in all three species (70% of aspen, 42% of lodgepole pine, 40% of jack pine). The second most abundant fatty acid in both pines was oleic acid while palmitic acid was the second most abundant in aspen. Abundance of the remaining fatty acids varied among species.

**Fig 3:**
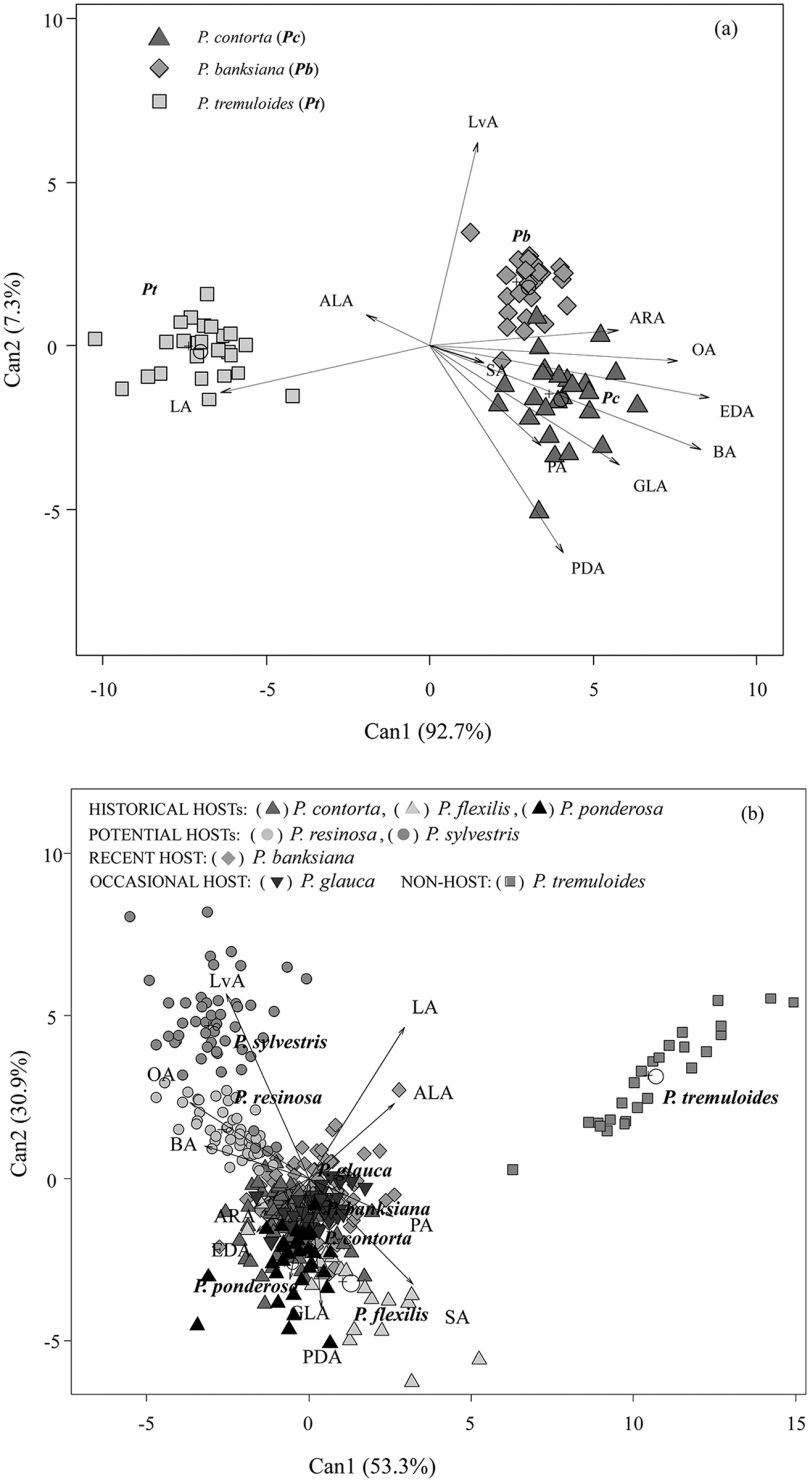
Canonical discriminant analysis biplot with Can1 and Can2 axes demonstrates a correspondence of fatty acid profiles of each tree species with individual centroids (+) based on the mean concentration of individual fatty acids. Points represent individual trees sampled. Vectors represent individual fatty acids (acronyms for individual fatty acids were shown in Table 1). **(A)** Among Pc (*Pinus contorta*), Pb (*P*. *banksiana*), and Pt (*Populus tremuloides*). All samples were collected in Alberta (n = 25). **(B)** Among historical (*P*. *contorta*, n = 90, *P*. *flexilis*, n = 22, P. ponderosa, n = 31), potential (*P*. *resinosa*, n = 49, *P*. *sylvestris*, n = 50), recent (*P*. *banksiana*, n = 122), occasional (*Picea glauca*, n = 41) hosts and non-host (*P*. *tremuloides*, n = 25).

Furthermore, canonical discriminant analysis showed the correspondence of fatty acid concentrations among lodgepole and jack pines and aspen (Fig 3A). Can1 and Can 2 axes explained 92.7 and 7.3% of the total variation respectively. Can1 axis discriminated both pines from aspen. The most discriminant variables along Can1 axis were linoleic and alpha-linolenic acids that were higher in aspen. Aspen showed lower abundance of oleic and behenic acids, compared to the two pine species. Along Can2 axis, the most discriminant variables were levulinic and pentadecanoic acids and both were slightly more abundant in both pine species.

When all eight tree species were included in canonical discriminant analysis, Can1 and Can2 explained 53.3 and 30.9% of the total variation respectively. Can1 separated aspen from all other conifer species (Fig 3B). Again, linoleic and alpha-linolenic acids were the most discriminant variables along Can1 axis and were higher in aspen than the other two pine species. Likewise, both oleic and behenic acids were less abundant in aspen. Along Can2 axis, the most discriminant variables were: 1) levulinic acid that was more abundant in scots pine; (2) stearic acid that was more abundant in limber pine; and (3) gamma-linolenic and pentadecanoic acids that both were higher in ponderosa pine. The remaining conifer species, including red, lodgepole, and jack pines, and white spruce, were clustered together. Additional comparisons among species were presented in Figs 1–3 and S1 File.

### Fungal growth

Our extraction and purification (solid phase extraction cartridge) method, confirmed by GC-MS, largely yielded free fatty acids and some triglycerides. Total purified lipid fractions of jack pine were suitable for *G*. *clavigera* while lodgepole pine and aspen were not. Follow-up analysis of the purified lipid fractions of lodgepole pine revealed higher amount of resin, which explains in part why we failed to observe any fungal growth. When we amended the media with the total saturated or unsaturated synthetic fatty acids representative of each tree species, *G*. *clavigera* grew only in the media amended with the total saturated fatty acids of jack pine.

**Fig 4:**
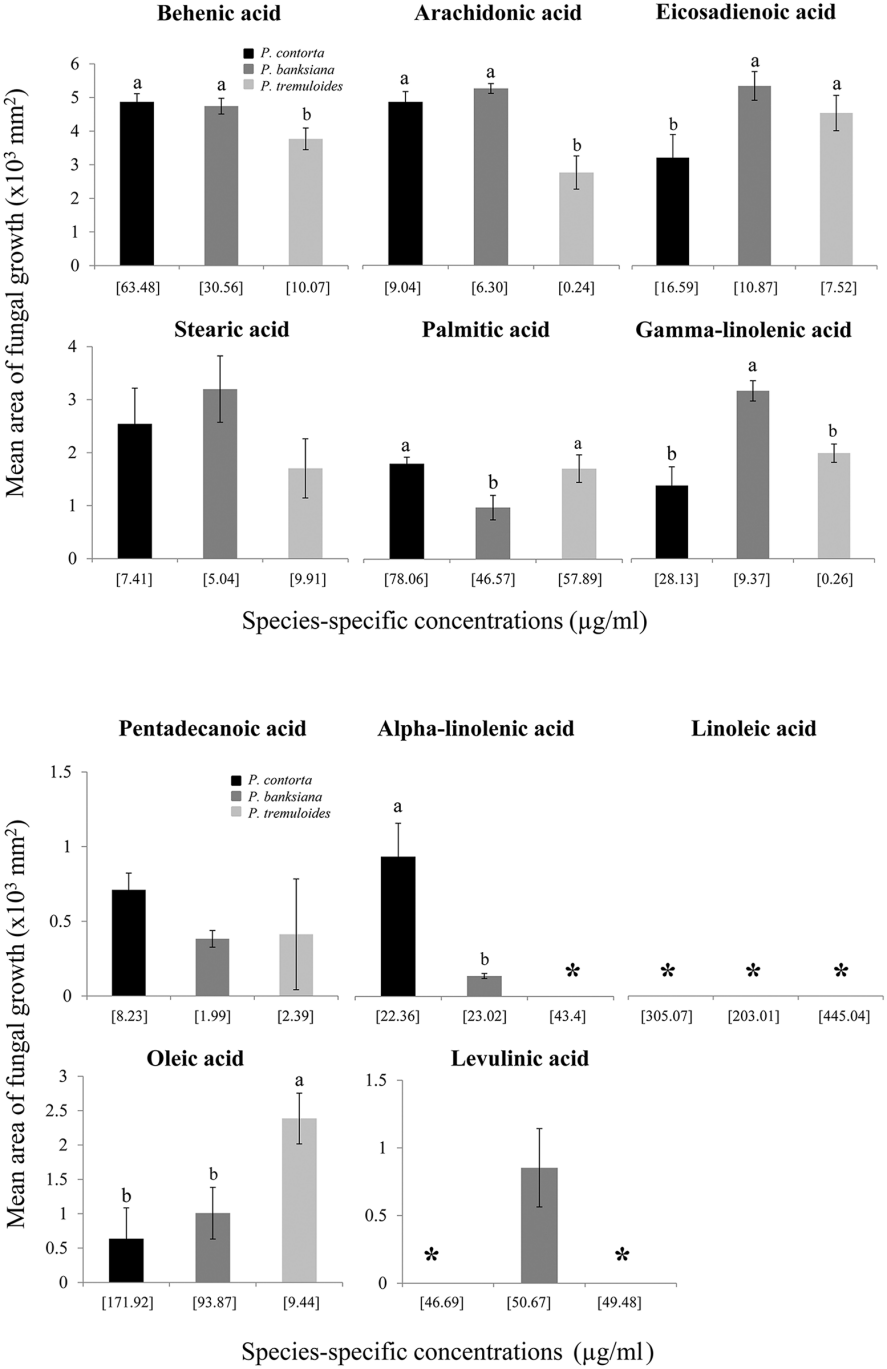
Mean (± SE) area of *Grosmannia clavigera* growth on the medium amended with the individual synthetic fatty acids at concentrations that simulate fatty acids of *P*. *contorta*, *P*. *bansksiana*, or *P*. *tremuloides* (n = 15). Total growth of the fungus was measured after 4 weeks in each Petri dish. The concentrations amended with the medium for each species were reported in S1 Table. ANOVA and Tukey HSD were applied for statistical comparisons. Different letters represent differences among tree species at α = 0.05.

When the individual fatty acids that simulated lodgepole pine, jack pine, or aspen were applied, differences in the *G*. *clavigera* growth were observed (Fig 4). Increasing concentrations of behenic (P = 0.01), palmitic (P = 0.009), and arachidonic (P = 0.001) acids increased the fungal growth, and lodgepole pine had the highest concentrations of all three fatty acids. Only levulinic acid at concentrations representative of jack pine yielded a fungal growth. Although increasing concentrations of stearic acid reduced fungal growth, it was not significant (P = 0.07). For behenic and arachidonic acids, both pines had similar fungal growth, whereas for palmitic acid, fungal growth was different among the two pine species. In contrast, increasing concentrations of oleic (P = 0.002), alpha-linolenic (P = 0.007), gamma-linolenic (P = 0.02), and eicosadienoic (P = 0.02) acids reduced fungal growth. Fungus did not grow at any concentration of linoleic acid and at the highest concentration of alpha-linolenic acid of aspen.

### Beetle larval survival

**Fig 5:**
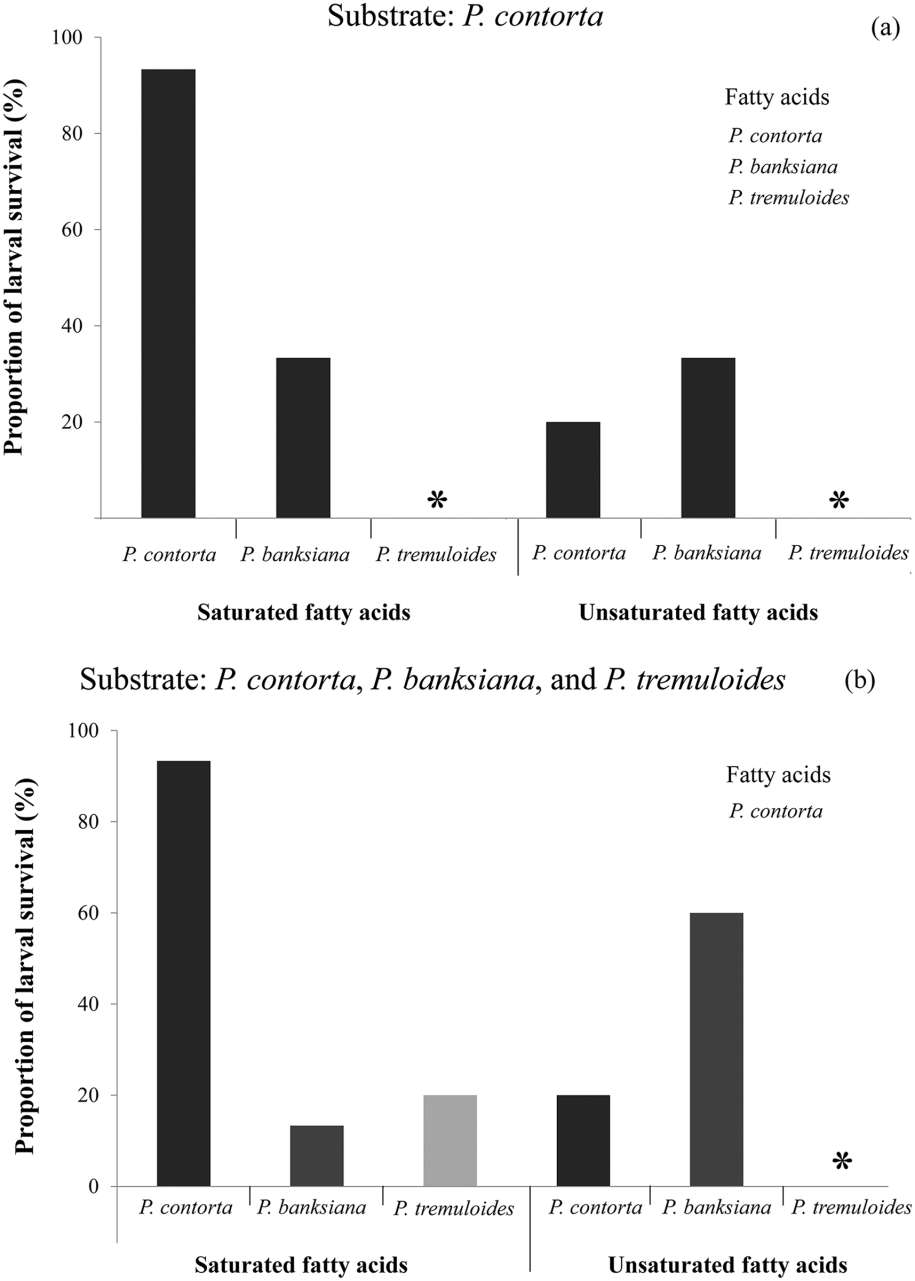
Proportion survival of mountain pine beetle (*Dendroctonus ponderosae*) larvae in rearing tubes amended with fatty acids. The concentrations amended in the tubes simulating each tree species were reported in S2 Table (n = 15). **(A)** Substrate of *Pinus contorta* was amended with the saturated or unsaturated fatty acids from *P*. *contorta*, *P*. *banksiana*, or *Populus tremuloides*. **(B)** Substrate of *P*. *contorta*, *P*. *banksiana*, or *P*. *tremuloides* were amended with the saturated or unsaturated fatty acids from *P*. *contorta*. SATFA indicates saturated fatty acid and UNSATFA unsaturated fatty acid. Stars represent the absence of larval survival.

Larvae continued their development when lodgepole pine substrate was amended only with the total saturated or unsaturated fatty acids representative of lodgepole pine or jack pine, not with aspen (Fig 5A). Between pine species, the lodgepole pine substrate amended with the total saturated fatty acids of lodgepole pine had the highest larval survival rate (P<0.0001). There was no difference when lodgepole pine substrate was amended with total unsaturated fatty acids of either pine species (P = 0.59).

When species-specific substrates of all three species were amended with the total saturated or unsaturated fatty acids representative of lodgepole pine, with the exception of the aspen substrate-unsaturated fatty acid combination, all combinations were suitable for the larvae. The highest larval survival rate was associated with the lodgepole pine substrate amended with saturated fatty acids (P<0.0001) and the jack pine substrate amended with unsaturated fatty acids (P<0.01) (Fig 5B). There was no difference in the proportion of larvae survived in the saturated fatty acids of jack pine or aspen (P = 0.86).

**Fig 6:**
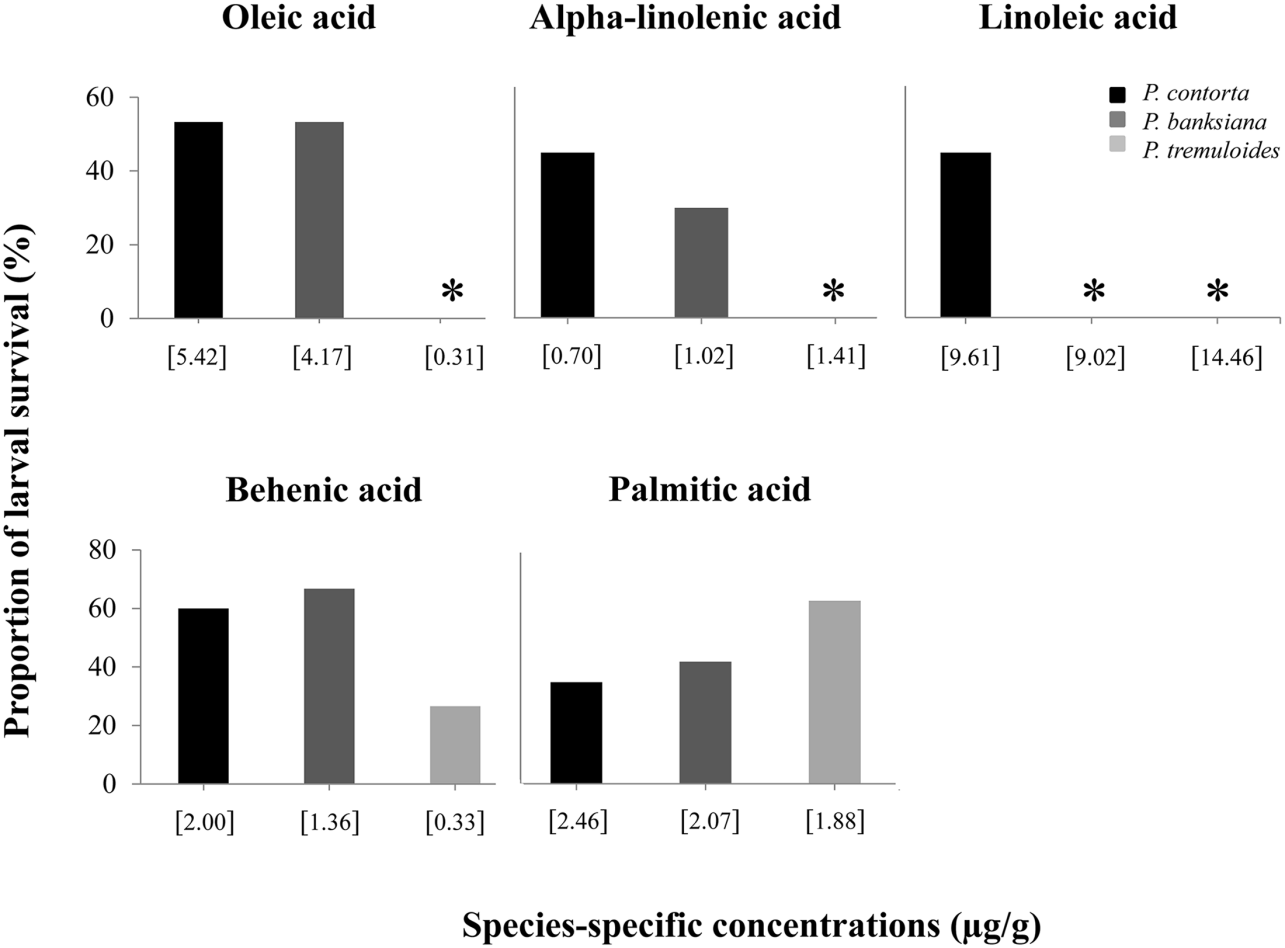
Proportion survival of the mountain pine beetle (*Dendroctonus ponderosae*) larvae in rearing tubes amended with individual fatty acids of *Pinus contorta*, *P*. *banksiana*, or *Populus tremuloides*. The concentrations amended in the tubes simulating each tree species were reported in S2 Table (n = 15).

We found opposing effects of individual fatty acids of tree species on larval survival (Fig 6). With the exception of palmitic and behenic acids, no larvae survived in the remaining three fatty acids of aspen. There was no difference between the two pine species for oleic (P = 0.99) and alpha-linolenic (P = 0.29) acids. Increasing concentration of behenic acids increased the proportion of larval survival by three-fold in both pine species relative to that of aspen and both lodgepole (P = 0.02) and jack (P = 0.02) pines were different from aspen. In contrast, increasing concentrations of palmitic, alpha-linolenic, and linoleic acids reduced larval survival. Among species, aspen had the highest concentrations of both alpha-linolenic and linoleic acids, and lodgepole pine had the highest concentration of palmitic acid. No larvae survived in alpha-linolenic acid from aspen and in linoleic acid from aspen and jack pine.

## Discussion

Plant fatty acids are some of essential nutrients for insect herbivores [40, 41], and thus their compatibility can be essential for insect herbivores. To our knowledge, our work provides the first direct evidence that tree fatty acids can affect the performance of both an insect and its fungal symbiont. We found that fatty acid composition of jack pine is suitable for colonization by MPB and its fungus. We provide two lines of evidence to support the suitability of jack pine fatty acids to MPB larvae and its symbiotic fungus. First, fatty acids of both jack pine and lodgepole pine had a similar effect on MPB larvae. Fatty acids have been found to be necessary for the development of insect herbivores [40, 41], and thus their compatibility has probably contributed to the colonization of jack pine by MPB. We demonstrated this using various combinations of the novel and historical hosts and aspen substrates amended with total or individual fatty acids at concentrations representative of lodgepole or jack pine. In contrast, fatty acids of aspen were incompatible with beetle larvae and substantially lowered larval survival even when they were mixed with the substrate of lodgepole pine. Particularly, no larvae survived in the substrate amended with alpha-linolenic acid of aspen, supporting the anti-feedant roles of this and other fatty acids on insects [42, 43]. Notably, the increased concentrations of behenic and oleic acids also increased the larval survival, and both historical and novel hosts had similar concentrations of these two fatty acids. These results may demonstrate concentration-dependent effects of fatty acids on larval survival, consistent with the observed effects of plant secondary compounds on bark beetles [44].

We propose two likely mechanisms to explain the importance of fatty acids on MPB larvae. First, incompatibility of the fatty acid content of diet (aspen) with beetles can have severe implications in MPB larvae as shown in several insect species, including reduction in mating, fertility, survival and production of unusual morphological abnormalities [45, 46]. Second, MPB larvae may lack the genes involved in the metabolism of incompatible fatty acids (e.g. high concentration of alpha-linolenic acid) as it has been shown in *Drosophila* flies that the transcription of genes involved in fatty acid metabolism was higher in the species resistant to the toxic levels of fatty acids relative to the non-resistant species [47]. The mechanism by which plant fatty acids affect bark beetle development at the cellular and molecular levels remains to be investigated.

Second, the growth of *G*. *clavigera* was similarly affected by the lipids and fatty acids of jack pine and lodgepole pine. It is worth noting that increasing concentrations of behenic and arachidonic acids also increased the fungal growth, and both historical and novel hosts had similar concentrations of these two fatty acids, suggesting that jack pine contains some fatty acids that enhance fungal growth at concentrations similar to those in lodgepole pine. In contrast, some lipids and fatty acids of aspen largely inhibited the fungal growth and particularly there was no fungal activity at alpha-linolenic acid of aspen. The anti-fungal properties of several fatty acids at high concentrations were demonstrated against plant pathogens [48, 49].

It is unknown how fatty acids—fungal interactions affect MPB larvae, but bark beetle-associated fungi including *G*. *clavigera* are involved in the breakdown of fatty acids [25, 28], and by doing so, may benefit larvae. In fact, in our preliminary trials, we could not rear beetles in the lodgepole pine substrate amended with fatty acids of any tree species without *G*. *clavigera* inoculation into the rearing tube. Apparently, fatty acids particularly linoleic and alpha-linolenic acids that are incompatible with the fungus at high concentrations are likely affecting membrane fluidity and protein activity in the fungus, as suggested for other fungal species [48, 49]. Considering the importance of fungal symbionts in bark beetle development, we hypothesize that the selection of host plants may be linked to the formation of associations with fungi that can grow therein, and may influence host selection and discrimination by bark beetles [8, 50].

In addition to these effects of fatty acids on MPB larva and fungus, canonical discriminant analysis on the fatty acid composition showed that jack pine was clustered together with lodgepole pine and other historical hosts of MPB, supporting earlier findings of similarities among these pine species based on their seed oil fatty acid composition [23]. Although total fatty acids of trees could be important, individual fatty acids are probably driving the suitability of host plants for insect colonization, as suggested for the plant secondary compounds [4, 9, 10, 11, 51]. For example, we found that, in addition to the several other fatty acids, both jack and lodgepole pines had similar, and lower, concentrations of linoleic and alpha-linolenic acids, compared to aspen. Interestingly, linoleic acid ranges from 44.4% to 55.9% in pine species in this study and others [22], whereas it comprises 75–80% of the total fatty acids in *Populus* species [21]. Linoleic and alpha-linolenic acids might be important in the evolution of bark beetle-host plant interactions and thus further studies are needed to determine if variation in concentrations of these two fatty acids among jack pine populations might alter tree suitability to MPB and its fungal symbionts.

In summary, we hypothesize that in addition to the plant secondary compounds [11], fatty acid profiles could also be used as a predictor of host plant suitability for insect and fungal colonization. Particularly, understanding of the relationship between the toxic effects of fatty acids and the insect herbivores and their microbial symbionts, is critical to determine the suitability of host plants. For example, canonical discriminant analysis clustered one of the potential hosts of MPB, red pine, together with the historical and novel hosts, suggesting that this species might be suitable to MPB colonization, supporting our results on suitability of this species [52].

## Supporting Information

S1 Fig Canonical discriminant analysis biplot with Can1 and Can2 axes demonstrates a correspondence of fatty acid profiles among host species with individual centroids (+) for each tree species based on concentrations of individual fatty acids (μg/g of dry weight of phloem). Each point characterizes the number of trees sampled for each host tree, including historical (*Pinus contorta*, n = 90, *P*. *flexilis*, n = 22, *P*. *ponderosa*, n = 31) and recent (*P*. *banksiana*, n = 122) hosts. Vectors represent individual fatty acids (Acronyms for individual fatty acids were shown in Table 1).
(TIF)

S2 Fig Canonical discriminant analysis biplot with Can1 and Can2 axes demonstrates a correspondence of fatty acid profiles among host trees with individual centroids (+) for each tree species based on concentrations of individual fatty acids (μg/g of dry weight of phloem). Each point characterizes the number of trees sampled for each host tree, including historical (*Pinus contorta*, n = 90, *P*. *flexilis*, n = 22, *P*. *ponderosa*, n = 31), potential (*P*. *resinosa*, n = 49, *P*. *sylvestris*, n = 50), and recent (*P*. *banksiana*, n = 122) hosts. Vectors represent individual fatty acids (acronyms for individual fatty acids were shown in Table 1).
(TIF)

S3 Fig Canonical discriminant analysis biplot with Can1 and Can2 axes demonstrates a correspondence of fatty acid profiles among hosts species with individual centroids (+) for each host species based on concentrations of individual fatty acids (μg/g of dry weight of phloem). Each point characterizes the number of trees sampled for each host species, historical (*Pinus contorta*, n = 90, *P*. *flexilis*, n = 22, *P*. *ponderosa*, n = 31), potential (*P*. *resinosa*, n = 49, *P*. *sylvestris*, n = 50), recent (*P*. *banksiana*, n = 122), and occasional (*Picea glauca*, n = 41) hosts. Vectors represent individual fatty acids (acronyms for individual fatty acids were shown in Table 1).
(TIF)

S1 File Supplementary Results. (DOCX)

S1 Table Mean concentration (μg/mL of wet weight of phloem) of individual fatty acids from *Pinus contorta*, *P*. *banksiana*, and *Populus tremuloides* added in each Petri dish plate to observe growth of a symbiotic fungus *Grosmannia clavigera* associated with *Dendroctonus ponderosae* in Fig 4. Acronyms for individual fatty acids were shown in Table 1.
(DOCX)

S2 Table Mean concentration (μg/mg of dry weight of phloem) of individual fatty acids from *Pinus contorta*, *P*. *banksiana*, and *Populus tremuloides* added in each tube to observe survival of *Dendroctonus ponderosae* larvae in Figs 5 and 6. Acronyms for individual fatty acids were shown in Table 1.
(DOCX)
