# Supplementary figures and images for: Fatty Acid Composition of Novel Host Jack Pine Do Not Prevent Host Acceptance and Colonization by the Invasive Mountain Pine Beetle and Its Symbiotic Fungus
Source: PLoS One. 2016 Sep 1;11(9):e0162046. doi: 10.1371/journal.pone.0162046 (PMC5008764; doi:10.1371/journal.pone.0162046)

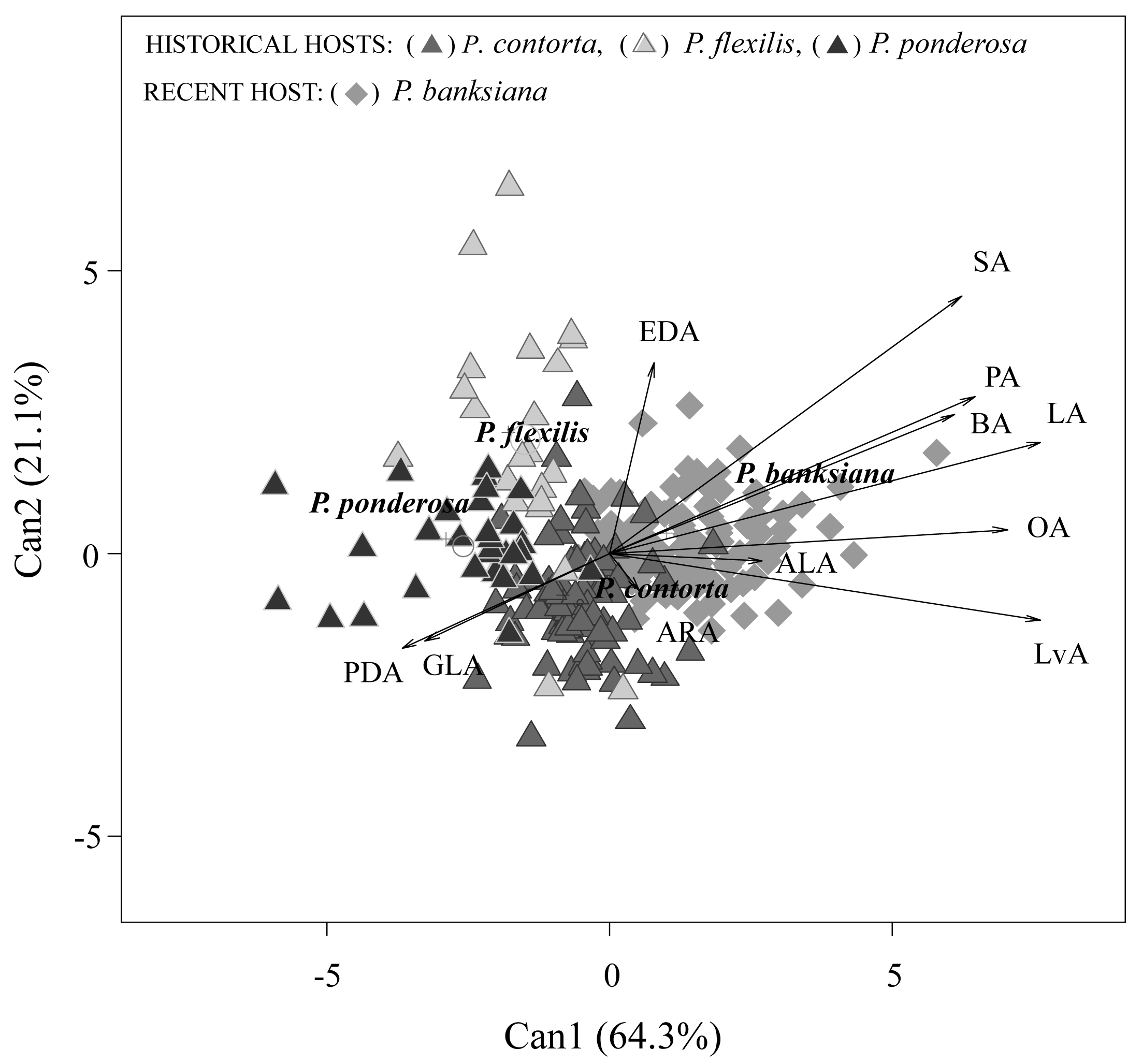

Supplement: S1 Fig — Each point characterizes the number of trees sampled for each host tree, including historical (Pinus contorta, n = 90, P. flexilis, n = 22, P. ponderosa, n = 31) and recent (P. banksiana, n = 122) hosts. Vectors represent individual fatty acids (Acronyms for individual fatty acids were shown in Table 1). (TIF) [file pone.0162046.s001.tif]

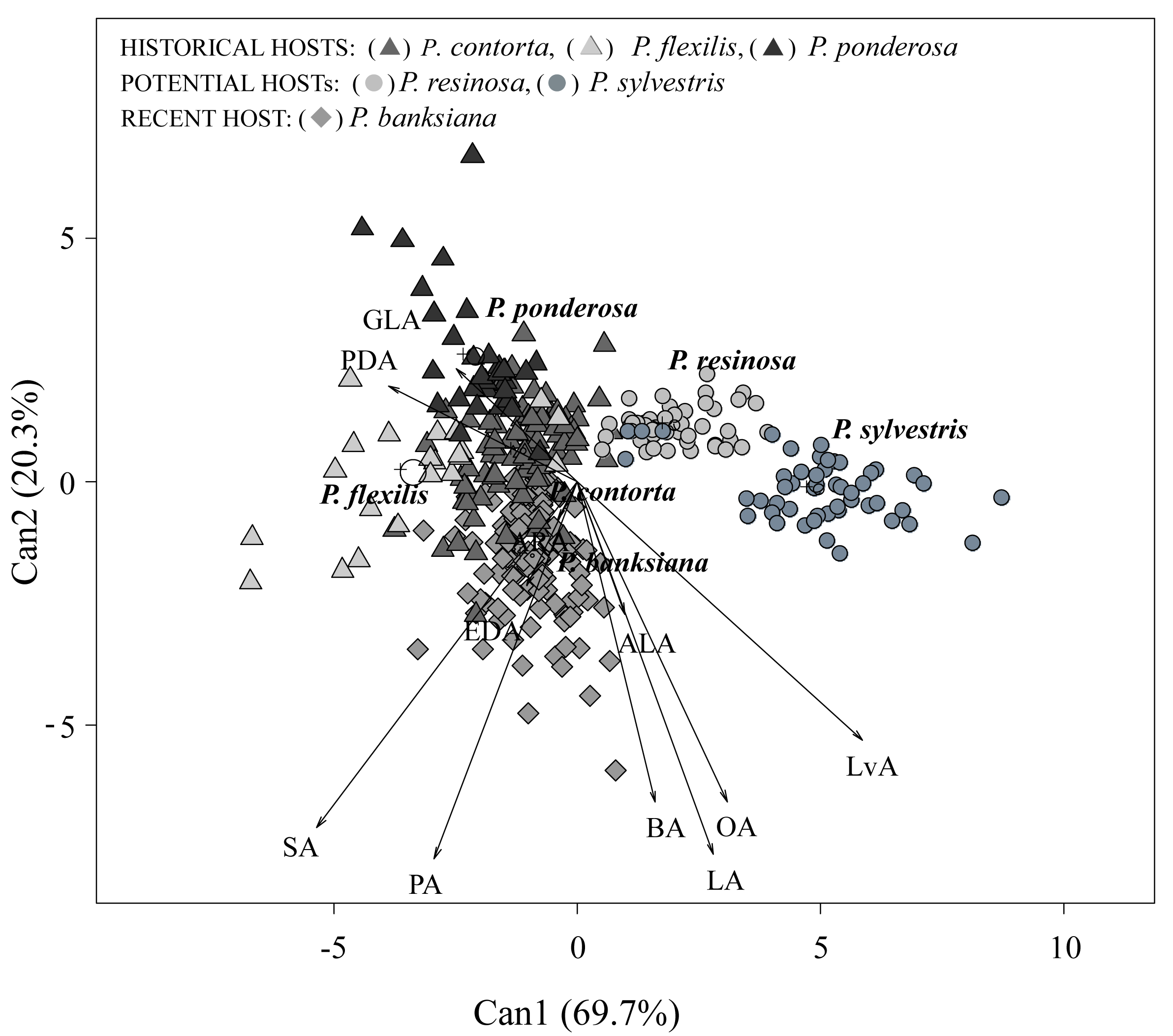

Supplement: S2 Fig — Each point characterizes the number of trees sampled for each host tree, including historical (Pinus contorta, n = 90, P. flexilis, n = 22, P. ponderosa, n = 31), potential (P. resinosa, n = 49, P. sylvestris, n = 50), and recent (P. banksiana, n = 122) hosts. Vectors represent individual fatty acids (acronyms for individual fatty acids were shown in Table 1). (TIF) [file pone.0162046.s002.tif]

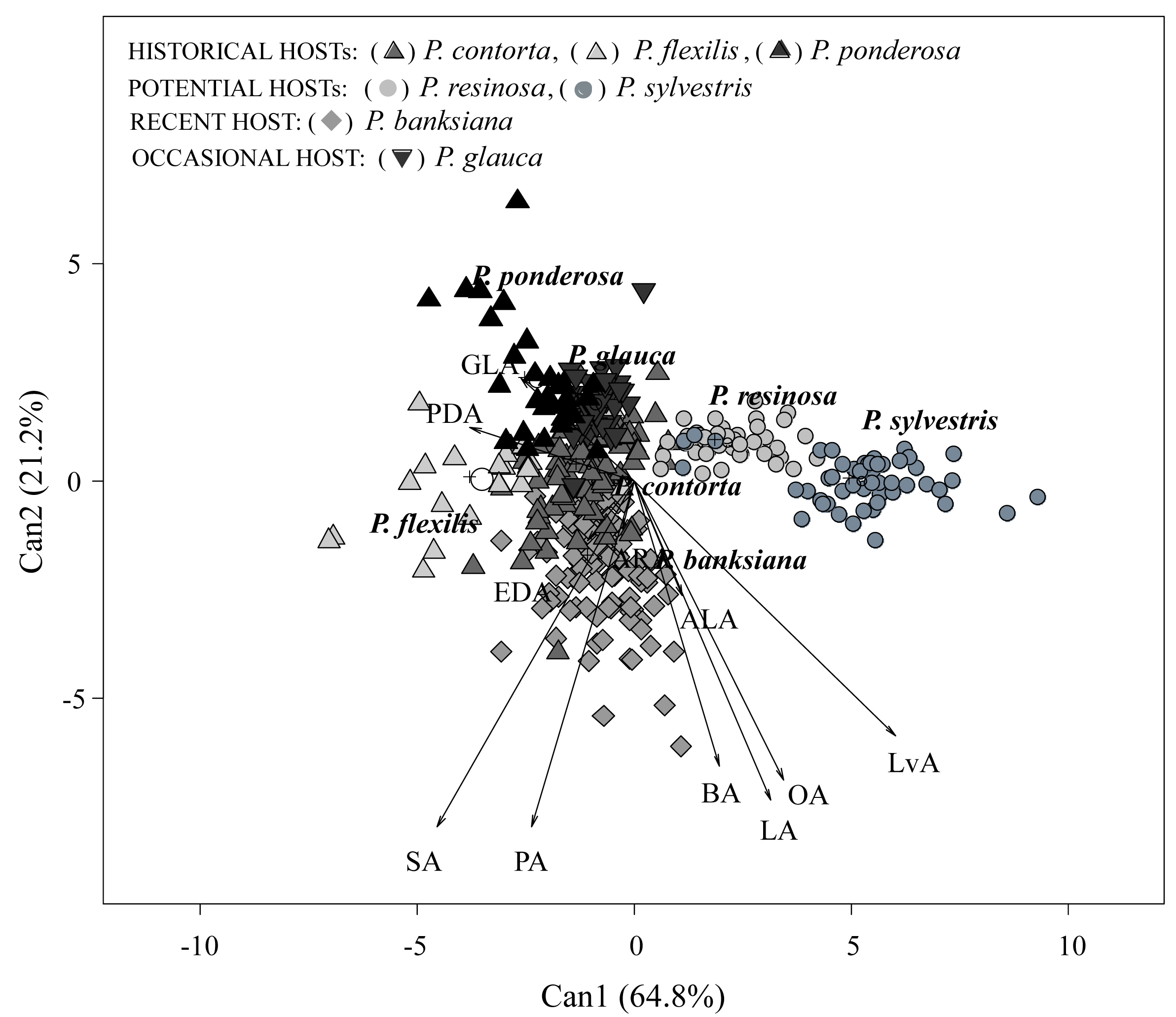

Supplement: S3 Fig — Each point characterizes the number of trees sampled for each host species, historical (Pinus contorta, n = 90, P. flexilis, n = 22, P. ponderosa, n = 31), potential (P. resinosa, n = 49, P. sylvestris, n = 50), recent (P. banksiana, n = 122), and occasional (Picea glauca, n = 41) hosts. Vectors represent individual fatty acids (acronyms for individual fatty acids were shown in Table 1). (TIF) [file pone.0162046.s003.tif]
