# Supplementary material for: Fatty Acid Composition of Novel Host Jack Pine Do Not Prevent Host Acceptance and Colonization by the Invasive Mountain Pine Beetle and Its Symbiotic Fungus
Source: PLoS One. 2016 Sep 1;11(9):e0162046. doi: 10.1371/journal.pone.0162046 (PMC5008764; doi:10.1371/journal.pone.0162046)
Supplement: S1 File — (DOCX) [file pone.0162046.s004.docx]

**Supplementary Results**

Additional canonical discriminant analyses were conducted among various combinations of eight tree species. We first analyzed historical (lodgepole, limber and ponderosa pines) and recent (jack pine) hosts and found that Can1 and Can2 explained 64.3 and 21.1% of the total variation (S1 Fig). Can1 discriminated jack pine from ponderosa pine. The most discriminant variables along Can1 axis were the pentadecanoic and gamma-linolenic acids that were higher in ponderosa pine. With the exception of eicosadienoic acid, the others were higher in jack pine. Along Can2 axis, the most discriminant variable was the eicosadienoic acid that was higher in limber pine.

In a separate canonical discriminant analysis, Can1 and Can2 explained 69.7 and 20.3% of the total variation among historical, recent and potential (red and scots pines) hosts (S2 Fig). Can1 separated limber pine from the two potential hosts, particularly scots pine. The most discriminant variable along Can1 axis was pentadecanoic acid that was higher in limber pine. Neither red or scots pine had any unique fatty acid that separated from others. Along Can2 axis, again ponderosa and jack pine separated and gamma-linolenic acids that were higher in ponderosa pine. With the exception of pentadecanoic acid, the others were higher in jack pine.

Furthermore, Can1 and Can2 explained 64.8 and 21.2% of the total variation among historical, potential, recent, and occasional (white spruce) hosts (S3 Fig). Can1 separated limber pine from the two potential hosts and pentadecanoic acid was the most discriminant variable along Ca1 axis. It was more abundant in limber pine than the two potential hosts. Along Can2 axis, ponderosa pine and white spruce were separated from jack and limber pines and with the exception of pentadecanoic and gamma-linolenic acids, other fatty acids were higher in the recent host.
