# Supplementary material for: Fatty Acid Composition of Novel Host Jack Pine Do Not Prevent Host Acceptance and Colonization by the Invasive Mountain Pine Beetle and Its Symbiotic Fungus
Source: PLoS One. 2016 Sep 1;11(9):e0162046. doi: 10.1371/journal.pone.0162046 (PMC5008764; doi:10.1371/journal.pone.0162046)
Supplement: S1 Table — Acronyms for individual fatty acids were shown in Table 1. (DOCX) [file pone.0162046.s005.docx]

S1 Table. Mean concentration (µg) of individual fatty acids from *Pinus contorta*, *P. banksiana*, and *Populus tremuloides* added in each Petri dish plate, which contains 20 mL of liquid agar, to observe growth of a symbiotic fungus *Grosmannia clavigera* associated with *Dendroctonus ponderosae* in Figure 4. Amounts were calculated based on the concentrations of individual fatty acids (µg/mL wet weight of phloem) in each species. Acronyms for individual fatty acids were shown in Table 1.

| Tree Species | Saturated Fatty Acids | | | | | Unsaturated Fatty Acids | | | | | |
| --- | --- | --- | --- | --- | --- | --- | --- | --- | --- | --- | --- |
|  | PA | BA | LvA | PDA | SA | LA | OA | GLA | ALA | EDA | ARA |
| P. contorta | 78.06 | 63.48 | 46.69 | 8.23 | 7.41 | 305.07 | 171.92 | 28.13 | 22.36 | 16.59 | 9.04 |
| P. banksiana | 46.57 | 30.56 | 50.67 | 1.99 | 5.04 | 203.01 | 93.87 | 9.37 | 23.02 | 10.87 | 6.30 |
| P. tremuloides | 57.89 | 10.07 | 49.48 | 2.39 | 9.91 | 445.04 | 9.442 | 0.26 | 43.40 | 7.52 | 0.24 |
