# Supplementary material for: Fatty Acid Composition of Novel Host Jack Pine Do Not Prevent Host Acceptance and Colonization by the Invasive Mountain Pine Beetle and Its Symbiotic Fungus
Source: PLoS One. 2016 Sep 1;11(9):e0162046. doi: 10.1371/journal.pone.0162046 (PMC5008764; doi:10.1371/journal.pone.0162046)
Supplement: S2 Table — Acronyms for individual fatty acids were shown in Table 1. (DOCX) [file pone.0162046.s006.docx]

S2 Table. Mean concentration (µg) of individual fatty acids from *Pinus contorta*, *P. banksiana*, and *Populus tremuloides* added in each tube, which contains 0.330-0.400 mg of a ground substrate, to observe survival of *Dendroctonus ponderosae* larvae in Figures 5 and 6. Amounts were calculated based on the concentrations of individual fatty acids (µg/mg dry weight of phloem) in each species. Acronyms for individual fatty acids were shown in Table 1.

| Tree Species | Saturated Fatty Acids | | | | | Unsaturated Fatty Acids | | | | | |
| --- | --- | --- | --- | --- | --- | --- | --- | --- | --- | --- | --- |
|  | PA | BA | LvA | PDA | SA | LA | OA | GLA | ALA | EDA | ARA |
| P. contorta | 2.46 | 2.00 | 1.47 | 0.26 | 0.23 | 9.61 | 5.42 | 0.89 | 0.70 | 0.52 | 0.28 |
| P. banksiana | 2.07 | 1.36 | 2.25 | 0.09 | 0.22 | 9.02 | 4.17 | 0.42 | 1.02 | 0.48 | 0.28 |
| P. tremuloides | 1.88 | 0.33 | 1.61 | 0.08 | 0.32 | 14.46 | 0.31 | 0.01 | 1.41 | 0.24 | 0.01 |
